# Comparative Efficacy and Safety of Anterograde vs. Retrograde Iodine Staining During Esophageal Chromoendoscopy: A Single-Center, Prospective, Parallel-Group, Randomized, Controlled, Single-Blind Trial

**DOI:** 10.3389/fmed.2021.764111

**Published:** 2021-11-25

**Authors:** Xu Tian, Wei Yang, Wei-Qing Chen

**Affiliations:** ^1^Nursing Department, Universitat Rovira i Virgili, Tarragona, Spain; ^2^Department of Gastroenterology, Chongqing University Cancer Hospital, Chongqing, China

**Keywords:** esophageal cancer, endoscopic screening, chromoendoscopy, iodine solution, staining

## Abstract

**Background and Aim:** Chromoendoscopy with iodine staining is an important diagnostic method for esophageal carcinomas or precancerous lesions. Unfortunately, iodine staining can be associated with numerous adverse events (AEs). We found that the starting position of spraying iodine solution is likely the main reason of causing AEs. We conducted this work to determine whether clinical outcomes from anterograde iodine staining were superior to those achieved after retrograde iodine staining.

**Methods:** A total of 134 subjects with a health risk appraisal flushing (HRA-F) score of >6 for esophageal cancer were randomly assigned to receive anterograde or retrograde iodine staining in the esophagus. The primary endpoints were the pain and the amount of iodine solution consumption. The secondary endpoints were iodine-staining effect, detection yield, and response to starch indicator.

**Results:** Nine patients suffered from pain and six patients revealed positive response to starch indicator in retrograde iodine-staining group; however, no patient reported pain (0/67) and all patients revealed a negative response to starch indicator in anterograde iodine-staining group. The amount of iodine solution consumption in anterograde iodine-staining group (4.97 mL) was significantly lower than that (6.23 mL) in retrograde iodine-staining group; however, the iodine-staining effect and detection yield were comparable between the two groups.

**Conclusions:** Anterograde iodine staining during Lugol chromoendoscopy appears to be as effective, but significantly safer than retrograde iodine staining.

## Introduction

It has been estimated that appropriately 6,04,100 new esophageal cancer cases and 5,44,076 deaths from esophageal cancer occurred in 2020 worldwide, which ranks sixth in incidence and eighth in cancer-related mortality (1). Patients with esophageal cancer continue to have a poor prognosis with a 5-year survival rate of <36.0% between 2000 and 2014 (2). In early-stage esophageal cancer, however, the 5-year survival rate is expected to increase significantly to 85.0% (3).

Early detection of esophageal cancer remains challenging. Early-stage esophageal cancer is underdiagnosed during gastric endoscopy under white light because symptoms are subtle in the early stage (4). So, chromoendoscopy was developed to facilitate accurate detection of early-stage esophageal cancer. Among the several chromoendoscopy techniques available, chromoendoscopy with iodine staining is currently the ideal technique for early-detecting superficial esophageal cancer (SEC) (5) because it is relatively easy to perform (6) and sensitive to detect esophageal dysplasia and carcinoma (7). However, chromoendoscopy with iodine solution is often associated with a higher risk of adverse iodine-related events (8). Previous works have not only determined the impact of various iodine concentrations on patients' discomfort (9), but also determined the safety and efficacy of various neutralizing solutions such as vitamin C solution (6) and sodium thiosulfate solution (STS) (10).

Conventionally, iodine solution was initially sprayed from the Z-line and gradually moved backward to a point about 20 cm from the upper incisors, which is named as retrograde iodine staining. Interestingly, patients reported fewer adverse events (AEs) when iodine solution was initially sprayed from a point about 20 cm from the upper incisors and gradually moved forward to the Z-line in our routine daily practice, which has been named as anterograde iodine staining by our team. However, this has not been previously studied. We therefore conducted this work to determine whether outcomes from anterograde iodine staining were superior to those achieved after retrograde iodine staining during chromoendoscopy in the esophagus.

## Materials and Methods

### Trial Design

This was a single-center, prospective, parallel-group, randomized, controlled, single-blind work comparing the efficacy and safety of anterograde vs. retrograde iodine staining during esophageal chromoendoscopy.

### Participants

Potentially eligible patients who admitted our endoscopic center were screened for the eligibility. Health risk appraisal flushing (HRA-F) score of >6 was employed as the inclusion criteria (11).

We also developed the following exclusion criteria: (a) pregnant or lactating female patients, (b) the presence of other serious diseases, such as severe liver or kidney diseases, malignant tumors, and alcoholism, (c) patients received chemoradiation, (d) the presence of bleeding, perforation, pyloric obstruction or cancer AEs or potential risks, (e) patients had a history of chromoendoscopy using iodine staining within 1 year, (f) patients could not express their main complaint or cooperate with this work, (g) patients had gastroesophageal reflux disease, reflux esophagitis, Barrett's esophagus, esophageal cancer, esophageal varices, or esophageal ulcers, (h) patients had biopsy contraindications, and (i) patients were allergic to iodine.

### Interventions

All patients who were admitted to our endoscopic center between January and June 2021 were assigned to receive white-light gastroscopy using PROCESSOR VP-4450HD (Fujifilm Co, Tokyo, Japan) or a CLV-290SL endoscope (Olympus Co., Tokyo, Japan). Endoscopic examinations were performed by four experienced endoscopists, all with more than 5,000 cases of experience in gastroscopy procedures. All patients were instructed to take dyclonine hydrochloride mucilage orally (Yangtze River Pharmaceutical Co LTD, China) 10 min before anesthesia gastroscopy to achieve topical pharyngeal anesthesia and to provide lubrication.

Patients were randomly allocated to either anterograde (group A) or retrograde (group B) iodine-staining group using a computerized random sequence. Following gastroscopy procedures with WLI, esophageal chromoendoscopy with 2% iodine solution was performed. We used a catheter (PW5L1; Olympus, Tokyo, Japan) to spray iodine solution until the esophageal mucosa was evenly dyed, and the subsequent color changes were observed. In anterograde iodine-staining group, iodine solution was initially sprayed from a point about 20 cm from the upper incisors and gradually moved forward to the Z-line (see Video 1). However, in retrograde iodine-staining group, iodine solution was initially sprayed from the Z-line and gradually moved backward to a point about 20 cm from the upper incisors. Apart from the starting position of spraying iodine solution, other regimes utilized in two groups were comparable. Iodine-staining effect was evaluated 2–3 min after iodine staining. All patients were continuously captured from the Z-line toward a point about 20 cm from the upper incisors. After gastroscopy examination, all patients were sprayed with 10 mL of 2.5% STS over the esophageal mucosa to neutralize the iodine solution.

After the iodine-staining procedure, no follow-up endoscopy was scheduled as a part of this work.

### Outcomes

The primary endpoints were the degree of pain and the amount of iodine solution consumption. A Wong–Baker Faces Pain Rating Scale was used to measure heartburn and retrosternal pain (0 = no pain at all, 1 = slight pain, 2 = faire pain, 3 = moderate pain, 4 = extreme pain, and 5 = worst pain imaginable) (12). Patients were informed before performing the gastroscopy procedure that the evaluation of pain and discomfort would be conducted immediately after the completion of iodine staining. Pain evaluation was performed when patients recovered after anesthesia. A score of 0 or 1 was defined as no pain, whereas a score of 2–5 was defined as pain. The amount of iodine solution consumption was calculated by the nurse who assisted the endoscopist to perform colonoscopy by recording the volume before and after spraying iodine during esophageal chromoendoscopy.

**Figure 1:**
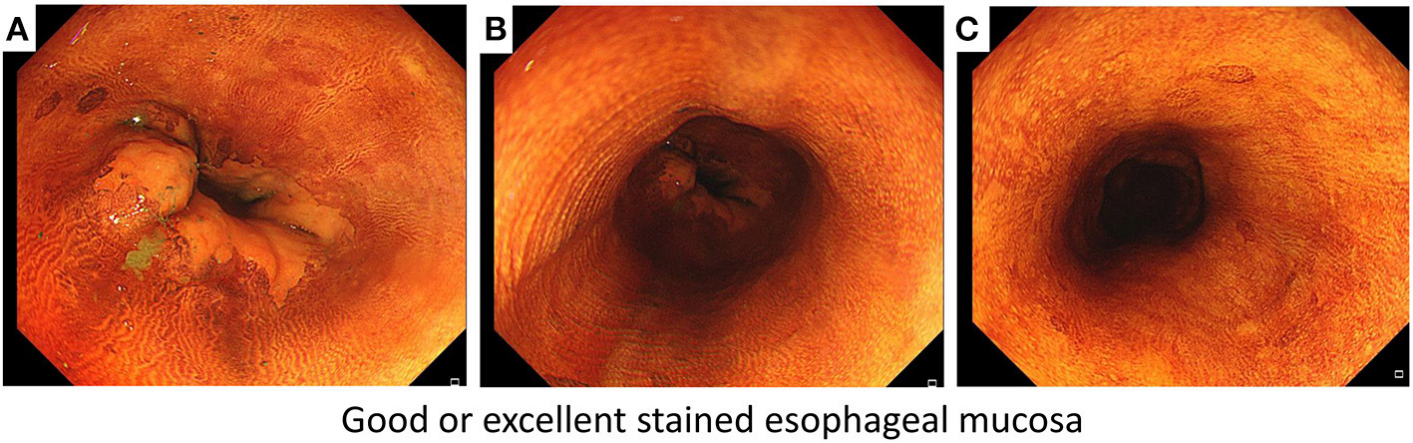
The criteria of defining good or excellent iodine-stained esophageal mucosa according to three points of targeted esophagus including **(A)** Z-line filed, **(B)** middle field of targeted esophagus, and **(C)** a point about 20 cm from the upper incisors.

**Figure 2:**
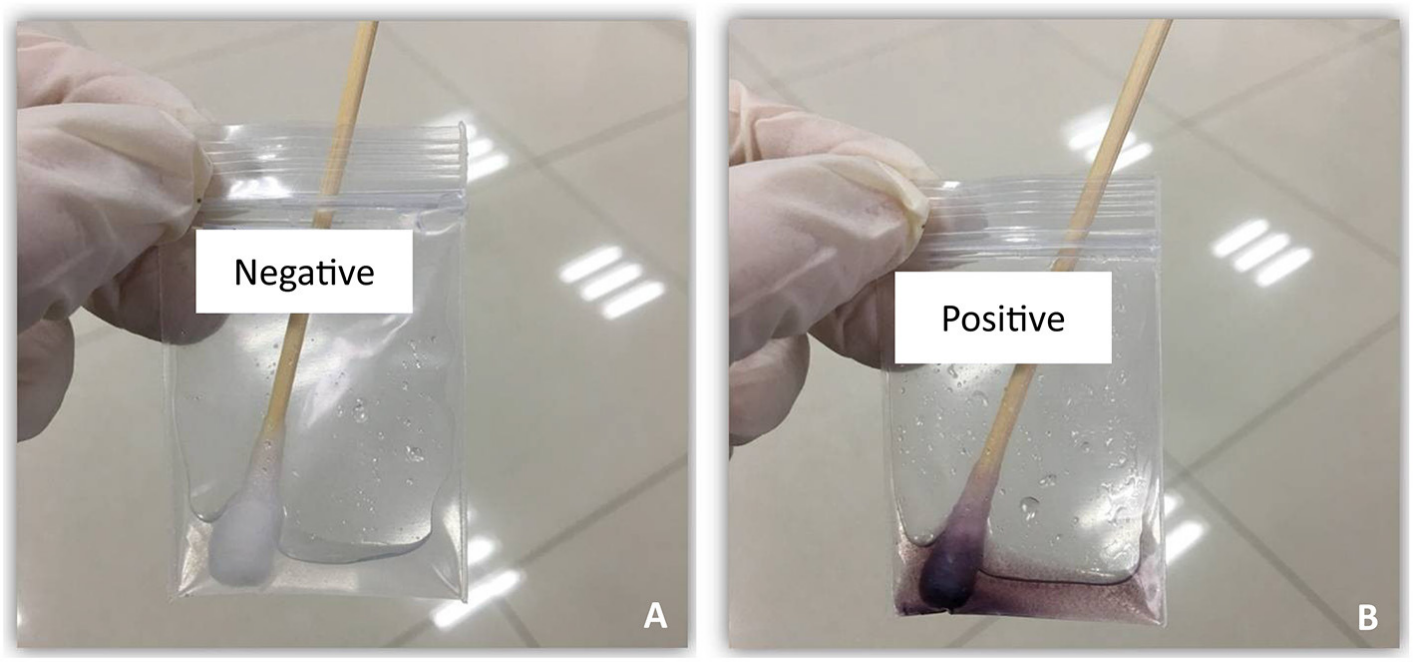
Starch indicator for negative **(A)** or positive **(B)** response.

The secondary endpoints were iodine-staining effect, detection yield, and response to starch indicator. Currently, there are no definitive criteria for evaluating the iodine-staining effect, and we therefore developed the criteria of defining good or excellent iodine-stained esophageal mucosa (Figure 1) according to the method introduced previously (9). We recorded the number of patients who achieved good or excellent iodine-stained esophageal mucosa after iodine solution was sprayed only once, and then, the iodine-staining effect was evaluated by calculating the proportion of patients who achieved good or excellent iodine-stained esophageal mucosa after iodine solution was sprayed only once. During gastroscopy, the number of suspicious lesions was recorded and a biopsy was carried out by the endoscopist. Biopsy specimens taken at the index endoscopy were fixed in 10% formaldehyde, embedded in paraffin, sectioned at 5 μm, and stained with hematoxylin and eosin (H&E). A pathologic diagnosis was rendered for each biopsy specimen by experienced pathologists who were blinded to all endoscopic findings, and discrepancies were adjudicated by consultation. For the purpose of determining the reflux of residual iodine solution in gastric cavity into the esophagus or oral cavity (including larynx), a starch indicator was introduced in this work. We firstly used a cotton swab to collect the pharyngeal contents, and then, it was put into a starch indicator solution. We judged that residual iodine solution reversely flowed back to the oral cavity if the starch indicator solution changed to be purple, which was defined as a positive response (Figure 2) (13, 14) and indicating a higher risk of results in AEs.

### Sample Size

According to a pilot work, to allow for up to a 20% dropout, 104 subjects were required overall to ensure 86 subjects completed this work (43 in each group), with an 80% power to detect a superiority margin of a 15% difference for pain between the two groups.

### Randomization

Eligible patients were randomly assigned at a 1:1 ratio to either the anterograde or retrograde iodine-staining group. The randomization consequence was generated by the independent research nurse based on SPSS software, and sealed envelopes were used to conceal the randomization consequence during the work. Patients were recruited by research nurse who explained the aim of work and instructed patients to sign consent before the procedure. Each research nurse opened the envelope immediately before starting the endoscopy examination, and the patient was blinded to the staining method allocated.

### Statistical Methods

Normally distributed continuous variables are presented as the mean ± the standard deviation (SD) and were compared using the Mann–Whitney *U* test. Non-normally distributed continuous variables were analyzed with the Kruskal–Wallis test. Categorical variables are presented as percentages and were calculated by the chi-squared test and the Fisher's exact test. Statistical analysis was conducted using SPSS 22.0 (IBM Corporation, Armonk, NY, USA). *p*-value < 0.05 indicates a significant difference.

### Ethical Concerns

The work was conducted in accordance with the principles of the Declaration of Helsinki. The institutional review board of Chongqing University Cancer Hospital approved the work. This work has also registered at the public platform, with a registration number of ChiCTR2100048789.

## Results

### Participants

**Figure 3:**
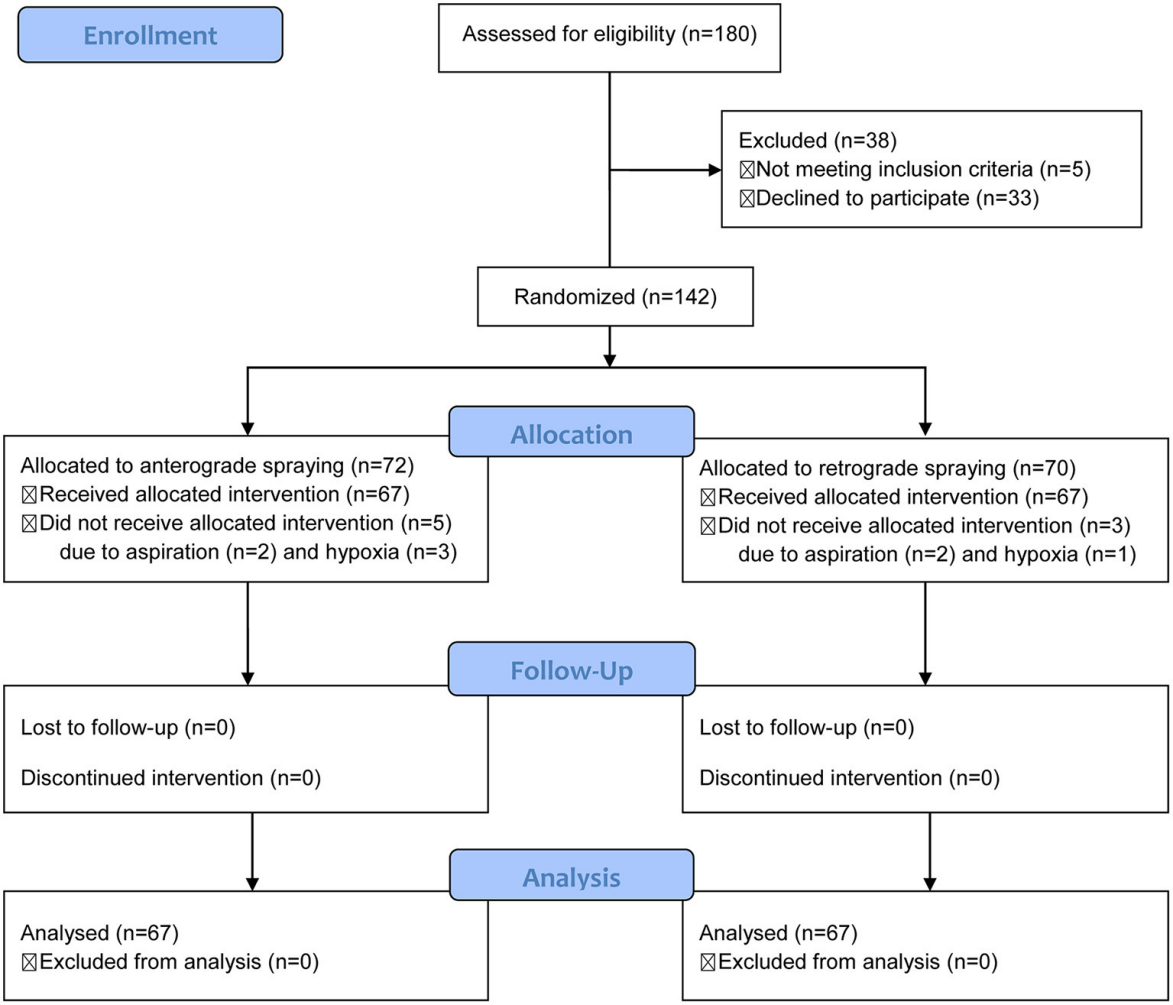
Consort diagram displaying recruitment and patient flow throughout the trial.

The consort flow diagram (Figure 3) documents the flow of recruitment and patient flow throughout the work. From January to June 2021, 180 patients were recruited, and 142 were included and then allocated to either the anterograde or retrograde iodine-staining group because of five patients did not meet inclusion criteria and 33 patients declined to participate in this work. A total of eight patients did not receive the allocated regimes including five in the anterograde iodine-staining group and three in the retrograde iodine-staining group because of aspiration (*n* = 4) and hypoxia (*n* = 4) (Figure 3). Baseline characteristics and procedure results were comparable in the two groups (Table 1).

**Table 1:** Characteristics of patients and procedure results.

**Variables**	**Group A (*n* = 67)**	**Group B (*n* = 67)**	***p*-value**
Age, years	56.48 ± 6.93	58.54 ± 7.97	0.11
Gender			0.47
Male	22	26	
Female	45	41	
Previous gastroscopy			0.22
No	38	32	
Yes	27	35	
BMI, kg/m^2^	24.41 ± 3.59	23.78 ± 3.14	0.28
Z-line, cm	37.97 ± 3.40	38.03 ± 2.75	0.91
Concomitant diseases			
DM	4	5	0.73
Hypertension	10	12	0.64
Cardiovascular disease	4	3	0.70
Pulmonary disease	3	2	0.65
Number of detected lesions, *n*	23	21	0.78
Histologically diagnosed neoplasia			
Squamous intraepithelial neoplasia, *n*	8	5	0.43

### Baseline Data and Endoscopic Procedure Characteristics

In both groups, most of the patients were women (anterograde iodine-staining group: 67.16% vs. retrograde iodine-staining group: 61.19%). The mean age was 56.48 and 58.54 years in anterograde and retrograde iodine-staining groups, respectively. The number of experiencing the previous colonoscopy, body mass index (BMI), the length of Z-line, and the distribution of concomitant diseases were comparable between the both groups.

Detection yield including the number of detected lesions (23 vs. 21, *P* = 0.78) and histologically diagnosed neoplasia (34.8 vs. 23.8%, *P* = 0.43) was comparable between the two groups (Table 1).

### Comparative Efficacy and Safety of Two Iodine-Staining Methods

**Figure 4:**
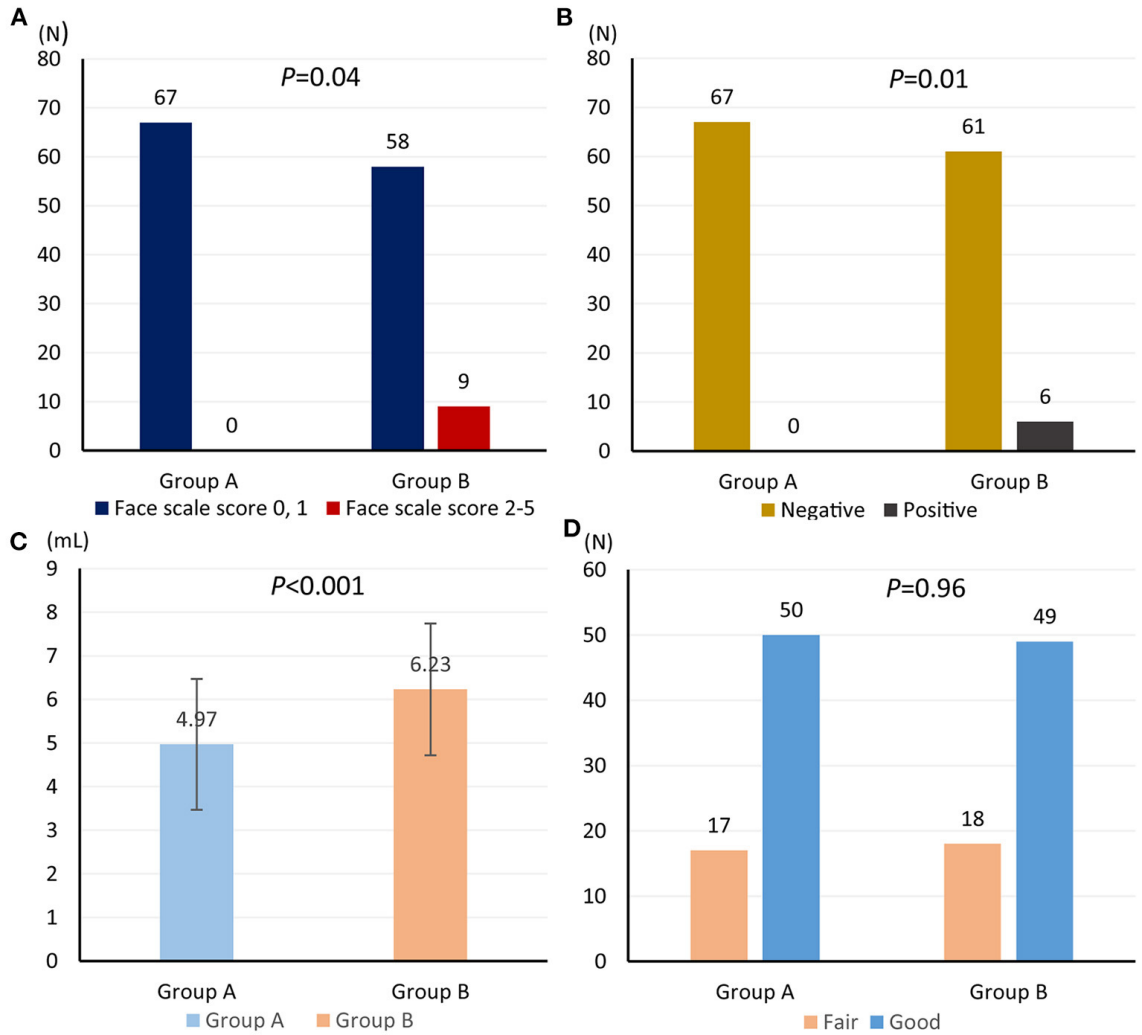
Comparison of face scale score for heartburn and retrosternal pain **(A)**, starch indicator **(B)**, amount of iodine solution consumption **(C)**, and iodine-staining effect **(D)** from anterograde and retrograde iodine staining.

Among 67 patients assigned in retrograde iodine-staining group, nine (13.4%) patients suffered from pain; however, no patient suffered from pain (0/67) in anterograde iodine-staining group (*P* = 0.04) (Figure 4A). Meanwhile, six patients in retrograde iodine-staining group indicated a positive response to starch indicator, which was more than that (0/67) in anterograde iodine-staining group (*P* = 0.01) (Figure 4B).

Amount of iodine solution consumption in anterograde iodine-staining group (mean volume, 4.97; 95% confidence interval (CI), 4.60–5.33) was significantly lower than that (mean volume, 6.23; 95% CI 5.86–6.60) in retrograde iodine-staining group (Figure 4C); however, iodine-staining effect was comparable between the two groups (95.5 vs. 97.0%, *P* = 0.65, Figure 4D).

## Discussion

Chromoendoscopy with iodine solution is undoubtedly useful for the early detection of superficial esophageal cancer (15); however, it can lead to painful and uncomfortable experiences because iodine solution can be associated with increased risk of several AEs, such as esophagitis, gastric ulcerations, and perforation (9). We find that the starting position of spraying iodine solution is closely associated with the risk of iodine-related AEs, without impairment in staining effect. However, it has not been previously studied. This work, which is the first randomized trial investigating the starting position of spraying iodine solution to date, suggests that anterograde iodine staining appears to be as effective, but significantly safer than retrograde iodine staining during esophageal chromoendoscopy. Compared with retrograde iodine staining, anterograde iodine staining obtains similar iodine-staining effect and detection yield. However, no patients who were assigned to receive anterograde iodine staining suffered from pain and revealed a positive response to starch indicator. Importantly, anterograde iodine staining consumes fewer amount of iodine solution compared with retrograde iodine staining.

The concentration of iodine solution is considered to be the major reason of causing several AEs during esophageal chromoendoscopy (4), and it is the fact that the reflux of residual iodine solution in the gastric cavity into the esophagus and oral cavity (including larynx) also plays a critical role in the occurrence of iodine-related AEs according to our daily experiences. In this work, anterograde iodine staining consumes fewer amount of iodine solution, and thus, the reflux of residual iodine solutions in the gastric cavity into the esophagus and oral cavity (including larynx) is almost impossible. As a result, fewer patients suffer from pain caused by iodine solution.

Some limitations should be interpreted. First, this work is a single-center analysis, and multicenter works are required to generalize our findings. Second, male patients have been reported to have a high risk of suffering from superficial esophageal cancer (16); however, more patients enrolled in our work were women, which may introduce selection bias. Third, we did not assess mucosal erosion of the bottom of the stomach by two staining methods. Fourth, we must also be acknowledged that our results could also be biased because of the univariate analytical methods used in our work could not separately investigate the impact of potential factors on results. Fifth, we did not investigate the impact of some confounders such as the COVID-19 pandemic and external stressors on the self-reported scale score for heartburn and retrosternal pain. These should be further investigated.

## Conclusions

In conclusion, we preliminarily speculate that anterograde iodine staining during esophageal chromoendoscopy appears to be as effective, but significantly safer than retrograde iodine staining. Anterograde as opposed to retrograde iodine staining could significantly reduce the amount of iodine solution consumption and the incidence of AEs, and also increase the compliance to the periodic endoscopy screening.

## Data Availability Statement

The raw data supporting the conclusions of this article will be made available by the authors, without undue reservation.

## Ethics Statement

The studies involving human participants were reviewed and approved by Institutional Review Board of Chongqing University Cancer Hospital. The patients/participants provided their written informed consent to participate in this study.

## Author Contributions

XT: investigation, formal analysis, methodology, writing, reviewing, and editing. WY: resources, formal analysis, writing, reviewing, and editing. W-QC: data curation, investigation, methodology, writing, reviewing, and editing.

## Funding

This work was partially supported by the Joint Growth Plan Project of Gastroenterologist & Hepatologist of China (approval number: GTCZ-2020-CX-50-0001).

## Conflict of Interest

The authors declare that the research was conducted in the absence of any commercial or financial relationships that could be construed as a potential conflict of interest.

## Publisher's Note

All claims expressed in this article are solely those of the authors and do not necessarily represent those of their affiliated organizations, or those of the publisher, the editors and the reviewers. Any product that may be evaluated in this article, or claim that may be made by its manufacturer, is not guaranteed or endorsed by the publisher.
